# Editorial to: Advance in the Treatment of Pediatric Leukemia

**DOI:** 10.3390/jcm11092361

**Published:** 2022-04-22

**Authors:** Rupert Handgretinger

**Affiliations:** 1 Department I–General Pediatrics, Hematology/Oncology, Children’s Hospital, University Hospital Tübingen, 72076 Tübingen, Germany; rupert.handgretinger@med.uni-tuebingen.de; 2 Abu Dhabi Stem Cell Center, Abu Dhabi, United Arab Emirates

The history of leukemia goes back many years and John Bennet, a Scottish physician, described in 1845 a 28-year old patient with swelling of the spleen who then developed fever, bleeding and increasing swellings in his neck, groin and armpits. The patient finally succumbed to this unknown disease. At autopsy, Bennet found a massive increase of white blood cells, which he interpreted as pus. However, he did not find a source of the pus, but nevertheless he called it a suppuration of blood. A few months later, the German pathologist Rudolf Virchow published a case report describing a patient in her mid-fifties whose white blood cells had overgrown her blood, and at autopsy, a milky white layer of white blood cells was seen without a microscope. Virchow knew of Bennet’s case, but did not agree with Bennet’s interpretation of the suppuration of blood, but rather wondered whether this was a disease of the blood itself. He named it in German “weisses Blut“ (white blood) but later changed it to the more academic-sounding word “Leukemia“ from leukos and haima, which means white and blood in Greek, respectively. In 1860, the first case of childhood leukemia was described by Biermer, a student of Virchow. A 5-year old girl became increasingly lethargic and developed skin bruises. Biermer found a high number of leukemic cells in her blood and the girl died within 3 days after Biermer’s diagnosis. In the next 100 years, no therapy was available for the affected children, and the mortality was 100%. When antifolates became available in the 1940ies, it was Sydney Farber who treated the first children with acute lymphoblastic leukemia (ALL) in 1947 with the antifolate aminopterin, with which he could induce at least temporary remissions [1]. In 1962, Danny Thomas founded the St. Jude Children Research Hospital in Memphis, USA, and the focus of the hospital at that time was the treatment of mainly lymphoblastic leukemia. Dr. Donald Pinkel, who was the first director of the hospital, initiated several consecutive clinical trials with modified regimes as new cytotoxic drugs became increasingly available. Given the combination of the different drugs, Dr. Pinkel called his studies “Total Therapy“, and in 1971, Pinkel and his colleagues published the first results of the total therapy [2]. Of 31 treated patients, 27 achieved remission, and the time to relapse was almost 5 years compared to the few months achieved by Farber. More importantly, 13 patients never experienced a relapse and in 1979, the St. Jude group reported on 639 patients treated in 8 consecutive total therapy studies, of which 278 patients had all treatment stopped after 2 ½ years of complete remission. Fifty-five of the 278 patients relapsed , mainly in the bone marrow. None of the 79 patients who remained in complete remission for at least 4 years off therapy have relapsed and ALL appeared curable in over one third of newly diagnosed patients who receive treatment for approximately 2 ½ years [3]. Dr. Pinkel stated in 1979 that ALL in children cannot be considered any more as an incurable disease and that palliation is no longer an acceptable approach to its initial treatment [4]. In parallel in 1969, Prof. Hans-Jörg Riehm and his colleagues in Germany initiated the West-Berlin Therapy study of ALL in which 8 drugs including Prednison, Vincristine, Daunorubicin, L-Asparaginase, Cyclophosphamide, Cytarabine, Methotrexate and prophylactic central nervous system (CNS) irradiation were applied until the patients’ tolerance limits. In 1977, Riehm et al. reported the 6-year experience of this approach on 73 children and adolescents. Six children died from therapy-related toxicity, 17 out of the 67 patients relapsed and 50 out of the 67 patients were in remission [5]. Based on these promising data, Prof. Riehm then introduced the concept of re-intensification in high risk patients and initiated the first cooperative ALL-BFM 76/79 studies in Germany, which initially comprised 3 centers (Berlin, Frankfurt, Münster) [6]. Currently, 115 German and international centers are participating in the most recent AIEOP-BFM ALL 2017 study. Through the cooperative trials in the US, Europe and many other countries, the cure rate of patients with ALL has increased with a 5-year overall survival (OS) rate exceeding 90% in high income countries. Based on the experience of the cooperative trials in ALL, similar trials were also initiated for the treatment of pediatric AML, JMML, and CML in children.

Since most of the children suffer from ALL, the focus of this special edition is mainly but not only on the advances in diagnosis, therapy, risk classification, clinical features, pharmacogenomics and new immunological approaches to the treatment of ALL, and new approaches to the therapy of AML, JMML and CML are also discussed. Finally, the indications for allogeneic transplantation and new transplantation approaches are presented.

Inaba and Pui start with advances in the diagnosis and treatment of pediatric acute leukemia. They describe the dramatic increase of the OS in the total therapy studies beginning with Total I-IV with an OS of 10% to 94% in the last Total XVI study. They describe in detail the cytotoxic drugs currently used and the medications used for molecular targeted therapy and for immunotherapy and discuss the classification of risk groups and the therapeutic approaches for the various genetic subtypes for acute pre-B-as well as for T-lymphoblastic leukemia. The authors emphasize the very important role of minimal residual disease (MRD), which has a major prognostic and therapeutic impact, and they point out that MRD levels, genetic classifications and clinical factors should be considered for risk stratification [7].

The biological and therapeutic implications of genomic alterations in ALL are discussed by Iacobucci, Kimura and Mullighan. They describe subtypes of ALL according to their specific genetic alterations, among them gross chromosomal abnormalities, transcription factor rearrangements and kinase alterations. They discuss in detail the gene expression signature for Ph-Like ALL, which comprises 10–15% in children. The correct diagnosis of Ph-Like ALL is important, since these patients may have targetable kinase alterations. In addition, they give a comprehensive genomic overview of T-ALL and its implication for diagnosis and treatment. The authors further discuss the value of the clinical implementation of high-throughput sequencing, including WTS (whole transcriptome sequencing, RNAseq), WGS (whole genome sequencing), WES (whole exome sequencing) and targeted DNA or RNA sequencing) for the detection of difficult subtypes of B- and T-ALL [8].

Although the cure rate is high, relapse of ALL is still the major reason for therapy failure. Most relapses occur in the bone marrow but can also occur in the CNS and testis, which are both considered to be sanctuary sites where chemotherapy is not effective. While in CNS and testes relapses, a specific local treatment together with systemic chemotherapy including irradiation, intrathecal therapy and orchiectomy and even allogeneic hematopoietic stem cell transplantation (HSCT) in high risk disease is necessary to induce long-term remission, much less is known on the outcome of children who have an non-CNS, non-testicular extramedullary relapse (other extramedullary relapse, OEMR). Lissat and colleagues have analyzed patients with OEMR who were treated in the multicenter ALL-REZ BFM trials between 1983 and 2015. Among 2323 patients, they identified 132 patients (5.6%) with OEMR. They describe in detail the different features and organ sites where the OEMR occurred and have classified OEMR into 5 subgroups. OEMR is more often seen in T-ALL compared to B-ALL, which is of prognostic relevance. The authors also give some guidance regarding the therapy of these patients, but also emphasize that based on the rareness of OEMR, international collaborations are necessary to prospectively evaluate the biology and treatment of his specific feature of ALL [9].

The intensive chemotherapy of ALL can come with severe organ toxicity, which can be life-threatening. In the beginning of the total therapies at Stjude, the patients often suffered from severe side effects, so that the fellows caring for the patients at that time called the total therapy among them “total hell“ (R. Handgretinger, own observation). However, tremendous progress has been made over the years in supportive therapies, but drug-specific toxicities still occur during therapy, including methotrexate-related encephalopathy, steroid-induced avascular bone necrosis, topoisomerase-II-associated secondary AML, and acute pancreatitis (AP) developing during treatment with L-Asparaginase. The important role of pharmacogenomic studies is demonstrated by Bartram and colleagues in AP, which is induced by L-Asparaginase. They conducted a genome-wide association (GWAS) study in 51 patients with AP and in 1388 patients without AP. They found single-nucleotide variants (SNVs) within the ABCC4 gene, which is an ABC transporter mediating the efflux of drugs and also is involved in the development of drug resistance. These findings emphasize the increasingly important role of pharmacogenomics, which might help in the future to identify patients at risk before they receive the therapy. The authors also emphasize that international joint efforts are needed to better assess genetic risks for AP and other rare toxicities based on GWAS studies [10].

Until more recently, chemotherapy and irradiation were the major pillars of the treatment of ALL. When patients became therapy-resistant, no other therapies were available, and most patients succumbed to their disease. With the introduction of the bispecific T-cell engaging (BiTE) antibody a decade ago, an immunotherapy became available which induced complete and MRD-negative remissions in chemoresistant patients. This new drug, now called Blinatumomab, activates T-lymphocytes which then attack and kill CD19-positive ALL blasts. Queudeville and Ebinger describe the introduction of Blinatumomab from the beginning until its approval by the authorities. They give a comprehensive review and summarize the various studies which have been and are currently being performed. They show that Blinatumab is finding more and more its way in frontline therapies rather than being used late in chemo-refractory patients. It is hoped that Blinatumomab might be able to replace some of the cytotoxic chemotherapy without compromising the OS and EFS. Therefore, the authors stress the fact that many questions are still open, such as the need for HSCT after remission induction by Blinatumomab, and that future clinical trials should reveal the role of Blinatumomab in frontline and relapse therapy [11].

Another way to activate T-cells against ALL blasts is the construction of artificial T-cell receptors. This technique uses the antigen-binding part of an antibody directed against targets on the ALL blasts in combination with additional costimulatory factors. T-cells are then genetically modified so that they express the antigen-binding part of the antibody on their surface, which makes them Chimeric Antigen Receptor (CAR) T-cells. Especially CAR T-cells directed against the CD19 antigen (CART19) have induced complete remissions in chemorefractory patients. Boettcher and colleagues present a very comprehensive overview on the development and biology of CAR T-cells for the treatment mainly but not only for ALL. They also discuss the side effects of CAR T-cell therapy, such as the Cytokine Release Syndrome (CRS) and the immune effector cell-associated neurotoxicity syndrome (ICANS), and point out new CAR constructs to improve the efficacy while reducing the side effects. The difference between Blinatumomab and CARs is the penetration of the CARs into the CNS and the long persistence in the patients, which is associated with a lower rate of relapse as long as the CAR T-cells are persisting. However, relapses can still occur, especially when the blasts lose the antigen, as it has been seen with CARs directed against the CD19 antigen. The authors review all current studies for the treatment of leukemia and also for solid pediatric tumors and discuss ways how to circumvent the antigen-negative relapses [12].

As in Blinatumomab and for CART19 cells, the CD19 antigen has been identified as an optimal target for immunotherapy of ALL with engineered anti-CD19 antibodies by Winterberg and colleagues. They developed an antibody fused to a single chain tumor necrosis factor (TNF)-related apoptosis-inducing ligand (TRAIL) domain. TRAIL was chosen because it induces apoptosis in malignant but not healthy cells. Indeed, the authors could demonstrate that this new antibody construct binds to ALL blasts and induces pronounced apoptosis in vitro and prolonged survival in mice transplanted with patients’ derived blasts. Interestingly, the combination of this construct with Venetoclax, which is an inhibitor of the anti-apoptotic protein BCL-2, induced synergistic apoptosis in vitro and in vivo in the mice models. These promising preclinical results warrant future preclinical and clinical studies [13].

The clinical outcome of other antibody conjugates is presented by Stokke and Bhojwani. For the treatment of ALL, the conjugate is composed of an antibody directed against CD22, which is, as CD19, almost universally expressed on ALL blasts. The antibody is conjugated to calicheamicin, a cytotoxic drug known as Inotuzumab ozogamicin. It has shown complete remission rates of 60–80% in patients with relapsed/refractory ALL. A similar construct comprised of an anti-CD33 antibody linked to calicheamicin (Gemtuzumab ozogamicin) is currently used for the treatment of AML. In addition, the authors give a comprehensive overview of other antibody/drug conjugates which are currently being studied in clinical trials for the treatment of ALL and AML and point out that the identification of optimal combinations with standard chemotherapy requires more clinical studies [14].

The current 5-year survival rates using intensive chemotherapy and also the new immunotherapies have only been achieved in high-income countries, and there is a great global disparity in treatment outcomes of ALL. Oh, Lee and Yeoh address this problem and show ways how to cure the curable patients with low-toxicity therapies in resource-limited countries (low-middle income countries, LMIC). They present data that for risk stratification, National Cancer Institute (NCI) standard risk criteria (age 1–10 years, WBC < 50,000 µL) are simple and effective. Depending on the available resources, other factors can be added. In LMIC, supportive care is also often limited, and the treatment-related morbidity and mortality can be more critical than relapses. Therefore, low-toxicity regimens should lead to improved OS. Since 80% of childhood ALL occurs in LMIC, the authors discuss the first steps to cure ALL in LMIC with less intensive therapy and less toxicity and with a better outcome, which could have a major impact on the 80% of children with ALL living in LMIC [15].

Progress has also been made in the treatment of patients with pediatric AML, which accounts for 15–20% of the pediatric leukemias with an incidence of approximately seven per million. Reinhardt, Antoniou and Waak give an overview of the past, present, and future and show the impressive progress, which has been made over the years. They discuss that this progress has been achieved by risk classification, CNS prophylaxis, introduction of MRD diagnostics, and the use of HSCT in high-risk patients. They also discuss the current cooperative trials and give an outlook on new therapies with targeted therapies and immunotherapies, including CAR T-cells [16].

Juvenile Myelomonocytic Leukemia (JMML) is a rare pediatric leukemia with shared features of myelodysplastic and myeloproliferative neoplasms. A common feature is the deregulation of the intracellular Ras signal transduction pathway. Mayerhofer, Niemeyer and Flotho present an overview of current treatment strategies. While HSCT is the only curative option for most patients, the authors describe a smaller proportion of children who survive long-term without transplantation. They review in detail the clinical and molecular risk factors which will give guidance to the therapy of this rare disease. The authors also point out experimental agents and targeted therapies, which might help to further improve the prognosis of patients with JMML [17].

Chronic Myeloid Leukemia (CML) is a clonal malignant disease characterized by the detection of the BCR-ABL1 fusion gene as a consequence of the t(9;22) reciprocal chromosomal translocation. Suttorp, Carrion and Hijiya give an overview of current treatment strategies. Since the current standard of care is the indefinite treatment with tyrosine kinase inhibitors (TKI), the humoral and cellular immune function might be reduced, and questions regarding the use of vaccines, including COVID-19 vaccines arise. The authors discuss the implication of TKI therapy for immunizations and for surveillance strategies and give guidance for the long-term care of these patients [18].

Finally, Algeri and colleagues discuss the role of HSCT in pediatric leukemia. Despite the remarkable achievements obtained with frontline therapies, transplantation is still for a number of patients the only curative approach. The authors discuss in detail the indications for HSCT for patients with ALL in first or second remission. In addition to some genetic factors, the MRD response has become an important indication for HSCT in first remission and in patients with late relapse. They then discuss the indication for HSCT in AML. Once again, genetic risk factors and well as MRD response will help to decide whether a patient needs an HSCT in first remission. All patients with a relapse of AML will have an indication for HSCT. The authors also discuss in detail the choice of the conditioning regimen and discuss the advantages and disadvantages of the various regimens. The detection of MRD pre-and/or post-transplant has become very important, and the authors discuss strategies for interventions using immunotherapy. Since not all patients will have a compatible donor, the authors also present data on alternative donors, including haploidentical donors. They stress the fact that, based on current outcome data, every patient in need of a transplant will have a donor. However, given the transplant-associated late effects, the determination of the appropriate role of HSCT in childhood leukemia remains a challenge [19].
